# The Effect of an Improved Environment According to Watson’s Theory of Human Care on Sleep, Anxiety, and Depression in Patients Undergoing Open Heart Surgery: A Randomized Controlled Trial

**DOI:** 10.3390/healthcare13020183

**Published:** 2025-01-18

**Authors:** Hatice Azizoğlu, Zeynep Gürkan, Yasemin Bozkurt, Canan Demir, Hatice Akaltun

**Affiliations:** 1 Faculty of Health Sciences, Nursing, Van Yüzüncü Yıl University, 65090 Van, Türkiye; haticeakaltun@yyu.edu.tr; 2 Institute of Health Sciences, Atatürk University, 25030 Erzurum, Türkiye; zeynepgurkan@yyu.edu.tr; 3 Cardiovascular Surgery Intensive Care Unit, Van Training and Research Hospital, 65000 Van, Türkiye; yaseminbozkurt350@gmail.com; 4 Health Services Vocational High School, Van Yüzüncü Yıl University, 65090 Van, Türkiye; canandemir@yyu.edu.tr

**Keywords:** anxiety, depression, improved environment, sleep quality

## Abstract

**Background/Objectives:** According to Watson’s Human Care Theory, an improved environment influences patients’ care processes. The purpose of this study was to examine the effect of an improved environment, according to Watson’s Human Care Theory, on sleep quality, anxiety, and depression in patients undergoing open heart surgery. **Methods:** Upon admission to the ward from the postoperative intensive care unit, the experimental group underwent environmental remediation for three days. The environmental arrangements ensured that the patient’s room maintained an appropriate temperature range of 18–26 °C and humidity values of 30–50%. Monitoring took place at 21:00, 22:00, and 23:00 on Days 1, 2, and 3, at which times, the brightness of the patient rooms gradually decreased. On the morning of the fourth day, the patients were interviewed face to face, and research questionnaires were filled out (ClinicalTrials.gov identifier of the manuscript: NCT06744023). **Results:** After the implementation of an improved environment in accordance with Watson’s Human Care Theory, the sleep duration of the patients in the experimental group (5.91 h) was higher than that of the control group (4.1 h). At the same time, the mean sleep quality score was measured as 300 ± 15.33 in the experimental group and 116.33 ± 14.94 in the control group. In addition, anxiety and depression levels were lower in the experimental group (5.63 ± 0.59; 4.53 ± 0.42) compared with the control group (12.03 ± 0.85; 10.03 ± 0.82). **Conclusions:** We recommend implementing improved environmental arrangements in accordance with Watson’s Human Care Theory to improve sleep quality and reduce anxiety and depression levels in patients undergoing open heart surgery.

## 1. Introduction

Worldwide, coronary artery disease (CAD) is the single most common cause of death. Unfortunately, more than seven million people die each year from CAD, which accounts for 12.8% of all deaths [1]. One of the most effective methods for treating CAD is coronary artery bypass grafting (CABG). Because of the invasiveness of this surgical intervention, patients experience several postoperative issues following CABG, including postoperative pain, anxiety, disturbed sleep patterns, changes in vital signs, and delayed recovery. After CABG, patient care should aim to reduce unwanted postoperative symptoms, increase patient comfort, and prevent complications [2,3].

Postoperative patient care should be carried out with effective nursing care planned in line with the patients’ needs [4]. A high-quality nursing care process should include not only invasive interventions but also care processes that address the patient in all aspects. Watson’s Human Care Theory (HCT) is based on nurses’ sincere, compassionate, and honest care behaviors, where nurses treat the individual in their care as cognitive, affective, and intuitive beings. At the same time, HCT aims to provide personalized and holistic care to individuals [5,6].

This theory, developed by Jean Watson between 1975 and 1979, defined nursing as effective caregiving. HCT is structured on three basic concepts. These concepts are “interpersonal care relationship, care situation/moment of care, and healing processes”. Healing processes constitute the main concept of HCT [6,7,8]. One of the nurses’ main tasks is to provide an improved environment for patients, which is defined as an area that supports and develops the individual’s natural healing capacity, fosters care relationships, and improves their environment during the healing process. Within the scope of an improved environment, light, art, architecture, aroma, air, music, sound, environmentally friendly green space, and nature are important factors [6,9]. Environmental interventions implemented in some institutional policies are used to improve psychological and physiological well-being [10]. It is reported in the literature that lighting, temperature, and humidity are environmental factors affecting sleep. In particular, it has been reported that light above 30 lux and inappropriate temperatures disrupt both slow wave and rapid eye movement (REM) sleep, while high humidity makes it difficult to stay asleep and disrupts slow wave sleep. 

Light is an environmental stimulus that interrupts the circadian rhythm, adversely affects sleep quality, and can cause nocturnal awakening [11,12,13,14]. Undesirable light exposure can lead to shifts in the circadian phase by interfering with the sleep onset phase. Exposure to evening light as low as 65 lux can shift the circadian phase by 1 h compared with exposure to evening light as low as 3 lux [11,15]. Hospital lighting has been reported to be 50–300 lux in ordinary patient rooms worldwide. However, it is reported that this range may be insufficient to regulate circadian rhythms [16]. Bernhofer et al. (2013) found that the mean night light exposure of the patients was 7.07 lux and that their sleep patterns were more continuous and less interrupted between the first and third days [16]. Giménez et al. (2017) conducted a study in a cardiology clinic to investigate how changing the illumination ratio of lights in a patient’s room affected sleep and found that patients in rooms illuminated with low light during the night experienced an increase in sleep time of 29 min or 7.3% compared with those in rooms with standard lighting [17]. In the study of Özgürsoy Uran et al. (2015), it was observed that the sleep pattern of a patient with heart failure changed after surgery; the patient woke up very frequently and did not wake up feeling rested, and daytime sleepiness increased. According to Watson’s HCT, it was determined that the patient’s sleep began to improve after nursing care interventions were applied [18].

However, during wakefulness and sleep, body temperature and skin temperature interact to maintain a balance between heat loss and heat production. Humidity is closely related to temperature. Patients’ rooms should be at 24 °C and 30% humidity in winter conditions and 24 °C and 50% humidity in summer conditions [19,20]. In reviews of the impact of temperature and humidity on sleep quality, there are a large number of high-quality studies using gold standard measures to characterize sleep disruption from controlled exposure to varying temperatures [11,21,22,23,24]. The results of these studies show a strong influence of temperature, humidity, and light load on sleep phases and suggest that the optimum design for the patients’ room environment should include a climate control that can maintain an ambient temperature between 17 and 28 °C with a relative humidity between 30% and 50% [11,19]. In a study by Yelden et al. (2015), noise, light, temperature, and humidity levels were monitored for three consecutive days in a neurological rehabilitation unit, and the measurements obtained were compared with the recommendations of the World Health Organization. In line with the findings of the study, it was concluded that patients were at risk for sleep disorders during their stay in the rehabilitation unit; sudden increases in noise, temperature, and light intensities may prevent a restful night’s sleep and may adversely affect the rehabilitation process due to impaired memory, learning, and well-being [22]. In Tian’s (2023) study, it is emphasized that all aspects of the physical space (sound environment, appearance/visual environment, decorative environment, smell, temperature, humidity, and ventilation) have important physiological and psychological effects on patients [25]. Based on all these, the light level of the patient’s room and the adjacent corridor should be kept in an appropriate lux range, and the humidity and temperature of the room should be kept in the optimum range in accordance with Watson’s HCT in order to keep the patient’s sleep pattern at an optimum level. 

At the same time, since open heart surgery involves major surgical interventions, the incidence of postoperative anxiety and depression is high, and changes in the quality of sleep of patients after surgery due to the effect of environmental factors also trigger the occurrence of anxiety and depression. Therefore, environmental improvements should be made to ensure sleep quality and help reduce anxiety and depression levels in the postoperative period of patients undergoing open heart surgery. The global number of deaths due to cardiovascular diseases increased from 12.4 million in 1990 to 19.8 million in 2022 due to metabolic, environmental and behavioral risks, together with environmental factors [26]. At the same time, cardiovascular disease remains a major cost burden for health services. In Europe, approximately 2500 patients are discharged per 100,000 inhabitants, costing EUR 106 billion annually, of which 49% go to inpatient hospital care [27]. It is thought that creating an improved environment in line with Watson’s HCT for the optimization of patients’ room conditions may accelerate the recovery process and help alleviate this burden by improving sleep quality and reducing anxiety and depression. Based on these factors, this study aimed to examine the effect of an improved environment implemented according to Watson’s HCT on sleep quality and anxiety and depression levels of patients undergoing open heart surgery.

### Research Hypotheses

**H1.**  *Environmental improvement, in line with Watson’s HCT, provided to patients undergoing open heart surgery has no effect on sleep quality, anxiety, and depression levels.*

**H2.**  *Environmental improvement, in line with Watson’s HCT, provided to patients undergoing open heart surgery has an effect on sleep quality, anxiety, and depression levels.*

## 2. Methods

### 2.1. Design

This study was conducted using a randomized controlled experimental research design and a single-blinded method. The ClinicalTrials.gov identifier of the manuscript is NCT06744023.

### 2.2. Setting and Sample

The study population consisted of patients who underwent open heart surgery between October 2023 and February 2024 in the cardiovascular surgery service of the Van Training and Research Hospital. Before collecting data for this study, the “G. Power-3.1.9.2” program calculated the sample size at an 80% confidence level. The “sample calculation formula with known universe (population volume)” (*n* = Nz2pq/d2 (N − 1) + z2pq) determined the sample. The literature review determined the “effect size” value to be 0.096. Taking a primary type error of 5% (Z = 1.96), a test power of 80%, and an effect size of 0.096 units, we calculated the minimum sample size as *n* = 60. Therefore, the study included 60 volunteer patients, with 30 placed in the experimental group and 30 in the control group [28,29,30].

*Inclusion criteria:* Patients who had undergone open heart surgery; were conscious, willing to communicate and cooperate; between the ages of 18–65; and had not been medically diagnosed with a mental illness.

### 2.3. Randomization

**Figure 1:**
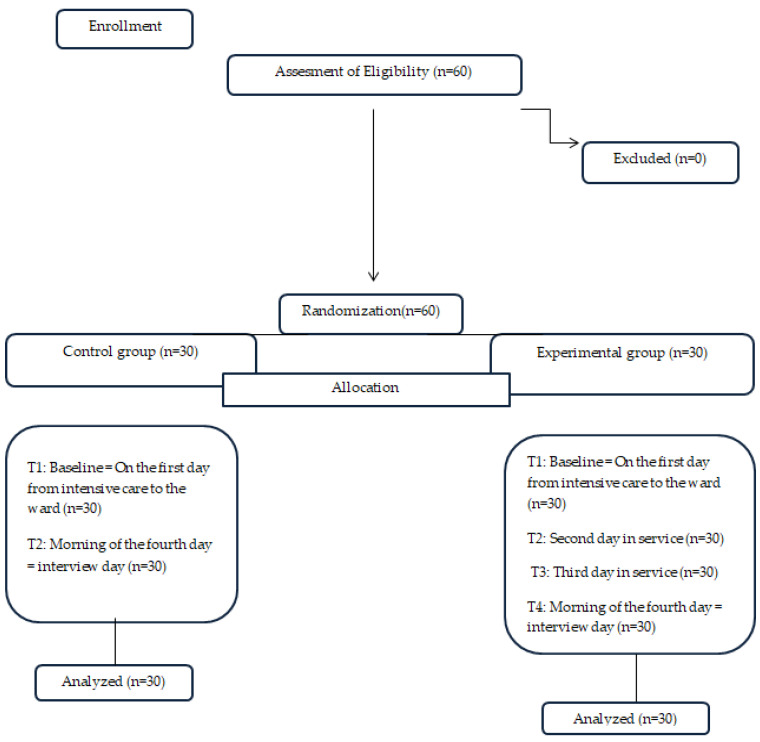
CONSORT diagram.

Clinical trials that compare two or more treatment or diagnostic methods are known as randomized controlled trials (RCTs) and are considered the gold standard of scientific evidence. The quality of RCTs is assessed according to CONSORT (Consolidated Standards for Reporting Trials) criteria. CONSORT is a 25-point protocol developed to report research results fully and clearly and to facilitate the reading and quality assessment of RCTs. This protocol provides standards on how to design, analyze, and interpret research. It also enables health professionals to critically assess the quality of the evidence obtained [31]. The study was conducted in accordance with CONSORT guidelines. Patients who met the research criteria were randomly divided into experimental and control groups according to the order of admission to surgery using the two-block randomization method on the Random.org website. Group assignment was determined by analyzing the sequence number of the table. This randomization technique was repeated until the required sample size was obtained for each group. Since the patients did not know which group they were in, a single-blinded method was used (Figure 1).

### 2.4. Instruments

Data were collected using the personal information form, the Richards–Campbell Sleep Questionnaire (RCSQ), and the Hospital Anxiety and Depression (HAD) Scale.

#### 2.4.1. Personal Information Form

The personal information form, which was prepared by the researchers by reviewing the literature, included 11 questions about the socio-demographic characteristics of the patients (gender, age, income status, educational status, etc.), history of surgery, history of chronic disease, and continuous medication use. This form also included a question about how many hours the patients slept the night before the interview with the researcher.

#### 2.4.2. Richards–Campbell Sleep Questionnaire (RCSQ)

The Richards–Campbell Sleep Questionnaire was developed by Richards in 1987 to assess the depth of nighttime sleep, the time it takes to fall asleep, the frequency of awakening, the duration of wakefulness, the quality of sleep, and the ambient noise level. A validity and reliability study of the Turkish adaptation of the scale was conducted by Karaman Özlü and Özer in 2015. Each item of the 6-item scale is evaluated on a scale ranging from 0 to 100 with the visual analog scale technique. A score of “0–25” indicates very poor sleep, and a score of “76–100” indicates very good sleep. The total score of the scale is evaluated over 5 items, and the 6th item evaluating the noise level in the environment is excluded from the total score evaluation. As the scale’s score increases, so does the patient’s sleep quality. In the Turkish validity and reliability study, Cronbach’s alpha coefficient of internal consistency was found to be α = 0.91 [32]. In this study, Item 6 was excluded from the total score evaluation, and Cronbach’s alpha coefficient of internal consistency of the scale was found to be α = 0.964. The Turkish version of the scale was used in this study.

#### 2.4.3. Hospital Anxiety and Depression (HAD) Scale

Zigmond and Snaith developed the HAD scale in 1983 to assess anxiety and depression in patients with physical illnesses. Aydemir et al. conducted the Turkish validity and reliability study of this scale in 1997. The scale, which includes anxiety and depression subscales, consists of 14 items. Responses are evaluated on a 4-point Likert scale and are scored between 0 and 3. However, the scoring of each item differs: Items 1, 3, 5, 6, 8, 10, 11, and 13 show gradually decreasing severity, with scores of 3, 2, 1, and 0. On the other hand, Items 2, 4, 7, 9, 12, and 14 are scored as 0, 1, 2, or 3 (Aydemir ve ark., 1997 [33]). The total scores of the subscales are obtained by summing these items’ scores. For the anxiety subscale, the 1st, 3rd, 5th, 7th, 7th, 9th, 11th, and 13th items are summed; for the depression subscale, the scores of the 2nd, 4th, 6th, 8th, 10th, 12th, and 14th items are summed. In Aydemir et al.’s study, Cronbach’s alpha coefficient of internal consistency for the anxiety subscale was found to be α = 0.8525, and it was α = 0.7784 for the depression subscale [33]. In this study, Cronbach’s alpha coefficient of internal consistency was α = 0.872 for the anxiety subscale and α = 0.835 for the depression subscale. The Turkish version of the scale was used in this study.

### 2.5. Intervention

Light is an environmental stimulus that adversely affects sleep quality by interrupting the circadian rhythm and may cause nocturnal awakenings [11,12,13,14]. Undesirable light exposure may lead to shifts in the circadian phase by preventing the sleep onset phase. Exposure to as low as 65 lux of evening light may shift the circadian phase by 1 h compared with exposure to 3 lux of evening light [11,15]. Worldwide, hospital lighting has been reported to be 50–300 lux in ordinary patients’ rooms. However, it is reported that this range may be insufficient to regulate circadian rhythms [16]. Therefore, in this study, the lux ranges of the patients’ rooms were reduced below 50 lux in the evening hours to regulate circadian rhythms in accordance with the literature. However, during wakefulness and sleep, body temperature and skin temperature interact to maintain a balance between heat loss and heat production. Humidity is closely related to temperature. Patients’ rooms should be at 24 °C and 30% humidity in winter conditions and 24 °C and 50% humidity in summer conditions [19,20]. Studies in the literature [11,21,22,23,24] show that temperature, humidity, and light load have a strong effect on sleep phases and state that the optimum design for the patients’ room environment should include climate control that can maintain the ambient temperature between 17 and 28 °C with relative humidity between 30% and 50% [11,19].

In this direction, patients who underwent open heart surgery and who were in the experimental group were admitted to the ward from the postoperative intensive care unit, and the ward environments were improved for three days according to Watson’s HCT. On the morning of the fourth day in the clinic, the patients were interviewed face to face, and the personal information form, RSCQ, and HAD scale were completed. The following improvements were implemented within the scope of environmental improvement.

- Considering the seasonal conditions, the patient’s room was ensured to be at an appropriate temperature (18–26 °C) and humidity (30–50%), and the temperature and humidity were gradually reduced and maintained in the appropriate range with monitoring at 21:00, 22:00, and 23:00 (Days 1, 2, and 3) (Table 1).
- In line with the environmental arrangements, the brightness of the lights in the patient’s room was gradually reduced at 21:00, 22:00, and 23:00 using the Light Meter LM-3000 app (Lightray Innovation GmbH, Zurich, Switzerland) (Days 1, 2, and 3) (Table 1).

### 2.6. Characteristics of the Measuring Instruments and Application

- The humidity and temperature of the patient’s room were measured with a digital-clock humidity meter and thermometer device. The digital-clock humidity meter and thermometer is a digital thermo-hygrometer that displays the indoor temperature and humidity values. The device can stand on a desktop or hang on a wall. The device displays temperature and humidity values, and it also stores the highest and lowest values in its memory. Temperature measurement units can be selected as 0 °C or 0 °F. The temperature measurement range is −10 to +600, and the accuracy is ±1 °C. The humidity measurement range of the device, with a temperature resolution of 0.1 °C, is between 10 and 99%. The humidity sensitivity is ±5%, and the humidity resolution rate is 1%. The device’s dimensions are (L) 102 × (W) 21 × (H) 110 mm. It weighs 122 g.
- The luminous intensity of the light in the patient’s room was measured with the Light Meter LM-3000 mobile application provided by the researcher. Luminous intensity is the amount of luminous flux per unit area of a surface per unit time, represented by E, in units of lux, and measured by a luxmeter. By definition, we divided the luminous flux of the surface by its area (E = lumen/m^2^). Light Meter LM-3000 is a mobile application that measures the illuminance of light in lux. Its measurement algorithm offers unrivaled precision to capture the slightest lighting changes, opening new possibilities in light pollution and health research. Developed by optical scientists and engineers and compatible with five languages, Light Meter LM-3000 is calibrated using professional Class A equipment for all iPhones and iPads.

The patients in the control group continued to receive standard treatment and care. On the morning of the fourth day after the patients were admitted to the ward from the postoperative intensive care unit, the patients were interviewed face to face, and the personal information form, RCSQ, and HAD scale were completed.

### 2.7. Data Assessment

Descriptive statistics for continuous variables were expressed as the mean and standard deviation, while categorical variables were expressed as numbers and percentages. The Kolmogorov–Smirnov test was used to assess the normality of the data distribution, while the Levene test assessed the homogeneity of variances. In terms of continuous variables, *t*-tests in independent groups were used in cases where normal distribution conditions were met, and Mann–Whitney U test statistics were used in cases where normal distribution conditions were not met in two independent group comparisons. To compare more than two groups independently using continuous variables, one-way analysis of variance (ANOVA) was used when the normal distribution condition was met. Kruskal–Wallis test statistics were used when the normal distribution condition was not met. In order to determine the relationship between continuous variables, the Pearson correlation coefficient was calculated when the normal distribution condition was met between the groups, and the Spearman rank correlation coefficient was calculated when the normal distribution condition was not met. The statistical significance level was taken as *p* < 0.05, and the SPSS statistical software version 19.0 (SPSS Inc., Chicago, IL, USA) package was used for the analyses.

## 3. Results

Analysis of the patients’ descriptive characteristics revealed that 80% of the experimental group’s patients had undergone open heart surgery with a diagnosis of CABG, 73.3% were male, 96.7% were married, 33.3% were literate, 63.3% had no job, 80% had never undergone surgery before, 60% had no chronic disease, and 50% had a history of continuous drug use (Table 2).

Analysis of the descriptive characteristics of the patients in the control group revealed that 66.7% had undergone open heart surgery with a diagnosis of CABG, 63.3% were male, 90% were married, 30% were primary school graduates, 26.7% had no job, 63.3% had never undergone surgery before, 50% had chronic diseases, and 66.7% had a history of continuous drug use (Table 2).

When the mean age of the patients was analyzed, it was found that the mean age of the patients in the experimental group was 60.60 ± 9.72 and the mean age of the patients in the control group was 59.43 ± 14.45. When the sleep duration of the patients the night before the interview was analyzed, it was determined that the patients in the experimental group slept 5.91 ± 1.58 h and the patients in the control group slept 4.10 ± 2.15 h. Upon analyzing the descriptive statistics and comparison results for age and sleep duration, we found that the difference between the experimental and control group averages was not significant for age but was statistically significant for sleep duration. Accordingly, the sleep duration of the patients in the experimental group at night before the interview was found to be higher on average than the sleep duration of the patients in the control group (*p* < 0.005) (Table 3).

**Figure 2:**
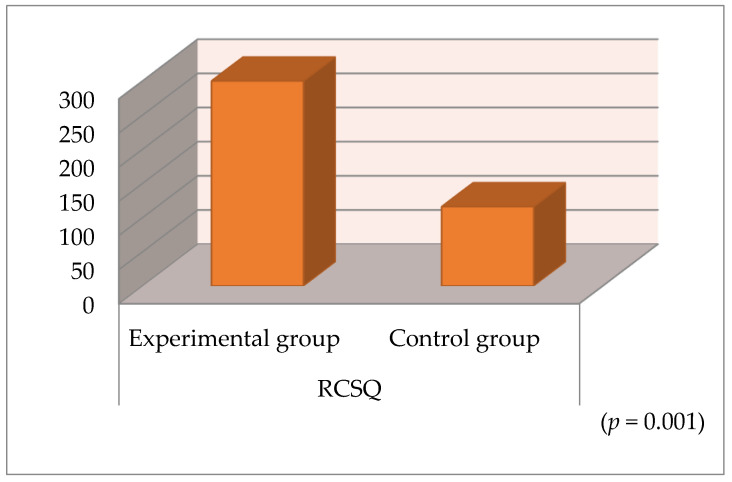
RCSQ scores in the experimental and control groups.

When comparing the mean RCSQ scores for the experimental and control groups, the difference between the means for the experimental and control groups was found to be statistically significant (*p* < 0.001). Accordingly, the mean RCSQ score was found to be significantly higher in the experimental group than in the control group (Table 4) (Figure 2). According to this, the sleep quality of the patients in the experimental group was higher than that of the patients in the control group.

**Figure 3:**
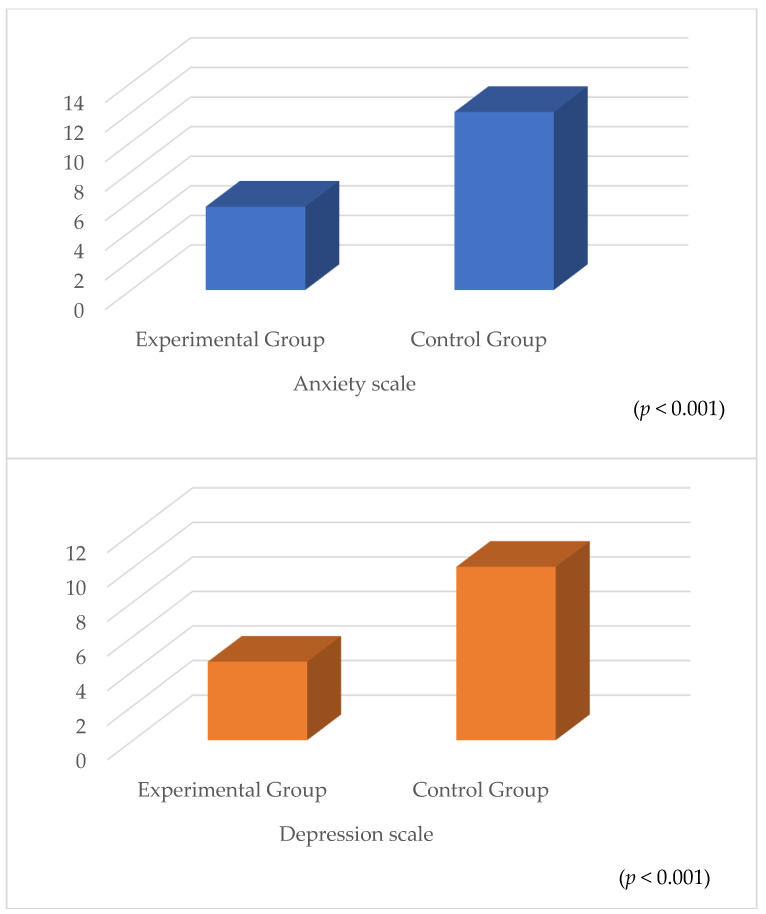
Anxiety and depression levels in the experimental and control groups.

The comparison results for the HAD scale in the experimental and control groups are given in Table 5. According to Table 5, the differences between the groups for both sub-dimensions of the HAD scale are statistically significant (*p* < 0.005). The experimental group’s mean was significantly lower than the control group’s. Accordingly, the anxiety and depression levels of the patients in the experimental group were lower compared with the patients in the control group (Table 5) (Figure 3).

Table 6 presents the correlations among sleep, depression, and anxiety scores in the experimental and control groups. In the experimental group, the relationship between sleep quality and both depression and anxiety was found to be statistically significant. The relationship between depression and anxiety was also found to be statistically significant (*p* < 0.05). Accordingly, a negative correlation was found between sleep and both depression and anxiety, and a strong positive correlation was found between depression and anxiety in the experimental group. In the control group, a strong positive correlation was found between anxiety and depression (*p* < 0.001) (Table 6).

## 4. Discussion

In this study, the sleep duration and sleep quality of the patients in the experimental group were found to be significantly higher than those of the patients in the control group, with the environmental organization applied in accordance with Watson’s HCT. In the literature, it is reported that the sleep time of patients can be improved with a limited lighting system used for night light. Giménez et al. (2017) conducted a study in a cardiology clinic to investigate how changing the illumination rate of the lights in a patient’s room affects sleep during hospitalization. Accordingly, Giménez et al. hospitalized patients in either a standardly illuminated room or a room with low light during the night. Upon analysis, it was found that patients in rooms illuminated with low light during the night experienced an increase in sleep duration of 29 min or 7.3% compared with those in rooms with standard lighting [17]. In the study of Özgürsoy Uran et al. (2015), it was observed that the sleep pattern of a patient with heart failure changed after surgery; the patient woke up very often, did not wake up feeling rested, and daytime sleepiness increased. Watson’s HCT determined that after applying nursing care interventions, the patient began to regulate their sleep [18]. In Talaz’s (2017) study, interviews were conducted with individuals with Type 2 diabetes based on Watson’s HCT, and data were collected qualitatively and quantitatively. As a result of the interviews, within the framework of quantitative and qualitative evaluations, it was determined that there was an improvement in the sleep quality of individuals [34]. Yelden et al.’s (2015) study monitored noise, light, temperature, and humidity levels for three consecutive days in a neurological rehabilitation unit and compared the obtained measurements with the World Health Organization’s recommendations. The study concluded, in line with the findings, that patients were at risk for sleep disorders during their stay in the rehabilitation unit; sudden increases in noise, temperature, and light intensities may prevent a restful night’s sleep and may negatively affect the rehabilitation process due to impaired memory, learning, and well-being [22]. In the study of Budiharjo et al. (2021), it was emphasized that one of the environmental factors affecting sleep quality was the temperature of the patient’s room, and it was concluded from the findings of the study that an increase in the ambient temperature above the optimal level negatively affected the sleep need and sleep quality of the patients [35]. Delaney et al. (2018), in 15 clinical units, aimed to investigate patients’ perceived sleep duration and quality and to determine the environmental factors associated with poor sleep reported by patients in the hospital; environmental noise, light, and temperature were monitored throughout the night. When the findings of the study were analyzed, the participants reported that the main factors associated with poor sleep were clinical care interventions and environmental noise. In the same study, 18% of the participating nurses stated that environmental temperature was one of the factors causing sleep disturbance in patients [36]. The findings of this study corroborate those found in the literature, demonstrating the effectiveness of these applications on sleep and factors related to sleep in various patient groups. Nursing practice has conducted numerous studies on sleep quality. However, it is thought that the results of planned studies based on nursing models or theories, which are the basic guidelines in nursing care, will be much more effective in nursing practice. At the same time, it is thought that the effect of environmental factors on sleep quality should be taken into consideration in patient care, and improving sleep quality may positively affect the healing process of patients. In addition, educating both patients and healthcare personnel about the effects of environmental factors on sleep may contribute to raising awareness about the relationship between environmental factors and sleep. In this way, a healthier sleep environment can be provided, and the healing process of patients can be positively affected.

In this study, the experimental group’s mean anxiety and depression scores were significantly lower than those of the control group. Serçe Yüksel’s (2022) study examined the impact of psychoeducation based on Watson’s HCT and self-compassion on the anxiety and depression levels of individuals diagnosed with hematological cancer. The intervention significantly reduced the patients’ anxiety and depression measurements [37]. In Seven’s (2018) study, the effect of care applied in line with Watson’s HCT on anxiety and depression in palliative care patients was examined, and in this direction, the comfort of the patients’ physical environment (light, temperature, sound, humidity, cleanliness, smell, etc.) was improved in the intervention group with the title “Supportive, protective, and/or healing environment”, and the suitability of the patients’ rooms in terms of oxygen and humidity was checked. The interventions led to a significant reduction in anxiety and depression levels in the intervention group compared with the control group [29]. In Tian’s (2023) study, it is emphasized that all aspects of the physical space (sound environment, appearance/visual environment, decorative environment, odor, temperature, humidity, and ventilation) have significant physiological and psychological impacts on patients [25]. The results of this study support the research results in the literature. The findings of closed-group studies in the literature [25,29,37,38] suggest that environmental interventions may be an effective approach in the management of anxiety and depression. Therefore, it is thought that health professionals should include not only medical interventions but also environmental factors in their treatment plans. Such an approach increases the psychological well-being of patients and helps to achieve more effective results in the treatment process. At the same time, functional aspects of design criteria are emphasized in most hospitals, but physiological and psychological factors are ignored. Appearance, lighting, temperature, humidity, sound, noise, and colors affect patients physiologically and psychologically. Therefore, it is thought that not only functional aspects but also physiological and psychological factors should be considered in hospital designs, and a healing environment based on Watson’s HCT should be created.

In this study, it was found that the anxiety and depression levels of patients in the experimental group, who received environmental improvement aligned with Watson’s HCT, decreased as their sleep quality increased. Nyer et al. (2013) found that participants with sleep disorders had higher rates of anxiety symptoms than those without sleep disorders [39]. In Demir and Sarıtaş’s (2022) study, the relationship among anxiety, stress levels, and sleep quality in patients after liver transplantation from living donors was investigated, and it was concluded that sleep quality decreased as anxiety levels increased [40]. Frontini et al. (2021) examined the relationship among anxiety levels, sleep, and physical activity during the COVID-19 quarantine and found that participants with higher sleep quality had lower anxiety levels [41]. Yin et al. (2023) examined the relationship between sleep quality and both anxiety and depression in elderly caregivers and concluded that there is a strong and inversely proportional relationship between sleep quality and both anxiety and depression [42]. The results of this study support the results of similar studies in the literature, and it is concluded that sleep quality, depression, and anxiety are conditions that share a complex and bidirectional relationship. At the same time, when the literature is examined, it is also mentioned that preoperative anxiety affects many components after surgery. It is concluded that anxiety in patients in the preoperative period has negative effects on sleep, comfort, and adaptation to environmental interventions in the postoperative period [43,44]. This study revealed that improving the environment in accordance with Watson’s HCT improved the sleep quality of the patients and decreased their anxiety and depression levels. This finding suggests that the environment plays an important role in the recovery process for patients. However, although many studies have examined the relationship among these three variables, there are no studies examining sleep quality, anxiety, and depression in open heart surgery by providing an improved environment in line with Watson’s HCT. Therefore, it is thought that studies with larger samples and studies based on more comprehensive methodologies should be conducted in open heart surgery. This approach aims to increase our understanding of the impact on patients’ sleep quality and psychological well-being and to facilitate the development of more effective health interventions.

## 5. Conclusions

Despite technological advances and the increase in the quality of surgical intervention, patients undergoing open heart surgery experience various difficulties and limitations, such as postoperative sleep disturbance, anxiety, and depression. The aim of nursing care for these patients should be to reduce unwanted postoperative symptoms, improve sleep quality, and reduce anxiety and depression levels. In this study, with the applied environmental improvement in line with Watson’s HCT, the brightness, humidity, and temperature of the patients’ rooms were regulated within appropriate ranges, and it was found that the patients in the experimental group had higher sleep duration and sleep quality and lower anxiety and depression levels compared with the patients in the control group. On the basis of the results of this study, it is recommended to implement improved environmental arrangements in line with Watson’s HCT to improve sleep quality and reduce anxiety and depression levels in patients undergoing open heart surgery.

### 5.1. Practice Implications

Since open heart surgery involves major surgical interventions, the incidence of postoperative anxiety and depression is high, and postoperative changes in patients’ routines, such as having to change their sleeping position, also affect their sleep quality. Therefore, environmental improvements should be made to ensure sleep quality in the postoperative period of patients who have undergone open heart surgery and to help reduce anxiety and depression levels. More clinical research is needed to create the best lighting, temperature, and humidity strategies to promote healing and well-being in healthcare institutions. The applicability of such research in the field of health services, as well as its effect on patients’ optimum sleep duration, is of great importance.

The findings of this study also emphasize the complexity and bidirectionality of the relationship between sleep quality and anxiety and depression. These findings provide an important basis for improving nurses’ caring practices and enhancing patients’ well-being.

### 5.2. Limitations 

This study is limited to patients who underwent open heart surgery in the Cardiovascular Surgery Clinic of a research hospital in eastern Turkey, met the sample selection criteria, and agreed to participate in the study. Therefore, the results can only be generalized to patients with the characteristics of this sample group. This study was conducted in a single center, but similar studies can be conducted in different regions or with samples consisting of patients with various socioeconomic and cultural backgrounds to increase generalizability in future studies. In addition, valid and reliable scales were used to assess sleep quality, anxiety, and depression levels in our study. However, the fact that these measurements are based on the subjective reports of individuals may limit the results. Therefore, in future studies, it is recommended that measurements such as anxiety, depression, and sleep quality should be evaluated on the basis of clinicians’ observations. At the same time, preoperative anxiety is an important confounder that also affects postoperative anxiety and depression levels. The fact that the preoperative anxiety level was not measured in this study is another limitation of the study. In future studies, preoperative anxiety levels can also be examined, and postoperative effects can be analyzed.

In addition to all these, in this study, the mean lumen intervals of the patients’ rooms were measured differently on the first, second, and third days. The intensity of external light sources, such as streetlights and moonlight coming from the windows in the patient’s room, may be different every night. The placement of objects in the patient’s room or the position of curtains can affect the reflection and absorption of light. Also, the sensitivity or battery level of the Light Meter LM-3000 app may cause variable results. In addition, fluctuations in the electrical network may cause slight changes in the brightness of light sources, bulbs may heat up differently over time, and light intensity may vary. In addition to all these factors, even if the same routine is followed every night, there may be small differences that are inadvertently overlooked (dimming time of the light source, switching on/off times, etc.), and people entering and leaving the room, devices used, or other activities that may affect the light measurement. In this direction, it is recommended to minimize external influences in the room during all measurements (for example, keeping the curtains closed), to calibrate the measuring device at the same point before each measurement, to standardize the room’s layout during the measurement, and to reduce the variability by taking more than one measurement at the same time each night and calculating the average of the measurements. 

The strengths of our study include the adoption of a randomized controlled design, the comparison of experimental and control groups, and the evaluation of the data obtained by statistical analysis using SPSS.

## Figures and Tables

**Table 1:** Lux range, temperature, and humidity measurements.

Day/h	Lux			Temperature			Humidity		
	Day 1	Day 2	Day 3	Day 1	Day 2	Day 3	Day 1	Day 2	Day 3
21:00	49.65 ± 17.33	35.66 ± 14.30	26.50 ± 10.59	24.94 ± 1.00	24.60 ± 1.19	24.28 ± 1.50	29.7 ± 0.87	28.59 ± 1.61	27.02 ± 1.54
22:00	29.76 ± 10.38	17.56 ± 8.38	8.83 ± 4.08	24.43 ± 1.25	24.15 ± 1.38	23.75 ± 1.60	28.37 ± 1.55	27.32 ± 1.45	25.79 ± 1.31
23:00	9.66 ± 5.52	5.66 ± 3.17	2.36 ± 1.61	24.31 ± 1.26	23.89 ± 1.56	23.47 ± 1.75	27.64 ± 1.50	26.67 ± 1.42	25.16 ± 1.36

**Table 2:** Demographic characteristics for the experimental and control groups.

Features	Experimental Group	Frequency	Percent (%)	Control Group	Frequency	Percent (%)
Diagnosis	CABG *	24	80	CABG	20	66.7
	MVR **	6	6	MVR	10	33.3
Gender	Male	22	73.3	Male	19	63.3
	Female	8	26.7	Female	11	36.7
Marital status	Married	29	96.7	Married	27	90
	Single	1	3.3	Single	3	10
Education status	Illiterate	6	20.0	Illiterate	6	20.0
	Literate	10	33.3	Literate	7	23.3
	Primary school	6	20.0	Primary school	9	30.0
	Middle school	3	10.0	Middle school	2	6.7
	High school	1	3.3	High school	3	10.0
	Undergraduate and graduate	4	13.3	Undergraduate and graduate	3	10.0
Profession	Not working	19	63.3	Not working	8	26.7
	Self-employment	3	10.0	Self-employment	6	20.0
	Officer	2	6.7	Officer	3	10.0
	Housewife	1	3.3	Housewife	7	23.3
	Other	5	16.7	Other	6	20.0
Previous surgery status	Yes	6	20.0	Yes	11	36.7
	No	24	80.0	No	19	63.3
Presence of chronic disease	Yes	12	40.0	Yes	15	50.0
	No	18	60.0	No	15	50.0
Presence of continuously used medication	Yes	15	50.0	Yes	20	66.7
	No	15	50.0	No	10	33.3

* Coronary artery bypass graft (CABG); ** mitral valve replacement (MVR).

**Table 3:** Descriptive statistics and comparison results.

	Group	*n*	Mean ± std. Deviation	*p*
Age	Experimental group	30	60.60 ± 9.72	0.715
	Control group	30	59.43 ± 14.45	
Total sleep hours on the 3rd night in the clinic	Experimental group	30	5.91 ± 1.58	0.001
	Control group	30	4.10 ± 2.15	

**Table 4:** RCSQ mean comparison results.

	Groups	*n*	Mean ± std. Error Mean	*p*
RCSQ	Experimental group	30	300 ± 15.33	0.001
	Control group	30	116.33 ± 14.94	

**Table 5:** Comparison results for the HAD scale in the experimental and control groups.

Groups		*n*	Mean ± std.	*p*
Anxiety	Experimental group	30	5.63 ± 0.59	0.001
	Control group	30	12.03 ± 0.85	
Depression	Experimental group	30	4.53 ± 0.42	0.001
	Control group	30	10.03 ± 0.82	

**Table 6:** Correlations among sleep, depression, and anxiety scores in the experimental and control groups.

Groups		Depression Total Score	RCSQ Total Score	Anxiety Total Score
Experimental group	Depression total score	1		
	RCSQ total score	r = −0.380 * (*p* = 0.039)	1	
	Anxiety total score	r = 0.720 ** (*p* = 0.001)	r = −0.363 * (*p* = 0.049)	1
Control group	Depression total score	1		
	RCSQ total score		1	
	Anxiety total score	r = 0.704 ** (*p* = 0.001)		1

*: Correlation is significant at the 0.05 level (2-tailed). **: Correlation is significant at the 0.01 level (2-tailed).

## Data Availability

The datasets used to support the findings of this study are available from the corresponding author upon request.
